# Supplementary material for: Effects of Plasma-Activated Water Treatment on the Inactivation of Microorganisms Present on Cherry Tomatoes and in Used Wash Solution
Source: Foods. 2023 Jun 23;12(13):2461. doi: 10.3390/foods12132461 (PMC10340167; doi:10.3390/foods12132461)
Supplement: Supplementary file 1 [file foods-12-02461-s001.zip › foods-2455976-supplementary.pdf]

foods

Supplementary Materials

Effects of Plasma-Activated Water Treatment on the Inactivation of Microorganisms Present on Cherry Tomatoes and in Used Wash Solution

Supplementary Figure S1:

Change of pH, oxidation-reduction potential (ORP) and electrical conductivity (EC) of PAW as a function of treatment time (min) with plasma.

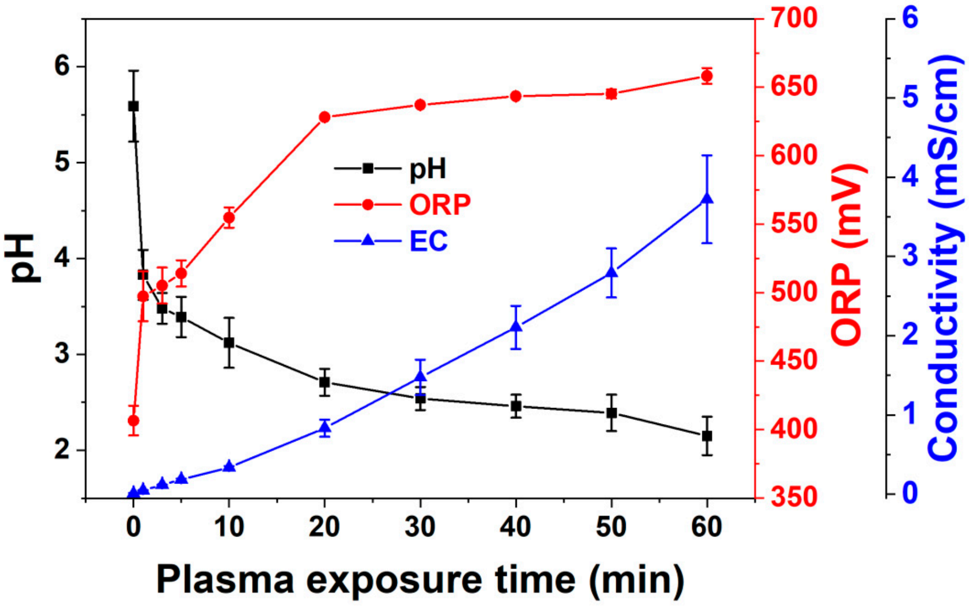

Foods **2023**, *12*, 2461. https://doi.org/10.3390/foods12132461 www.mdpi.com/journal/foods
